# Magnetic Resonance Imaging Features of Normal Thyroid Parenchyma and Incidental Diffuse Thyroid Disease: A Single-Center Study

**DOI:** 10.3389/fendo.2018.00746

**Published:** 2018-12-06

**Authors:** Taewoo Kang, Dong Wook Kim, Yoo Jin Lee, Young Jun Cho, Soo Jin Jung, Ha Kyoung Park, Tae Kwun Ha, Do Hun Kim, Ji Sun Park, Sung Ho Moon, Ki Jung Ahn, Hye Jin Baek

**Affiliations:** ^1^Department of Surgery (Busan Cancer Center), Pusan National University Hospital, Pusan National University College of Medicine, Busan, South Korea; ^2^Department of Radiology, Busan Paik Hospital, Inje University College of Medicine, Busan, South Korea; ^3^Department of Pathology, Busan Paik Hospital, Inje University College of Medicine, Busan, South Korea; ^4^Department of General Surgery, Busan Paik Hospital, Inje University College of Medicine, Busan, South Korea; ^5^Department of Otorhinolaryngology-Head and Neck Surgery, Busan Paik Hospital, Inje University College of Medicine, Busan, South Korea; ^6^Department of Nuclear Medicine, Busan Paik Hospital, Inje University College of Medicine, Busan, South Korea; ^7^Department of Anesthesiology and Pain Medicine, Busan Paik Hospital, Inje University College of Medicine, Busan, South Korea; ^8^Department of Radiation Oncology, Busan Paik Hospital, Inje University College of Medicine, Busan, South Korea; ^9^Department of Radiology, Gyeongsang National University School of Medicine and Gyeongsang National University Changwon Hospital, Changwon, South Korea

**Keywords:** thyroid, diffuse thyroid disease, autoimmune, Hashimoto thyroiditis, magnetic resonance imaging

## Abstract

**Background:** No previous studies have investigated the feasibility of magnetic resonance imaging (MRI) diagnosis for detecting incidental diffuse thyroid disease (DTD). This study investigated MRI features of normal thyroid parenchyma and incidental DTD.

**Methods:** From January 2008 to December 2017, 387 patients underwent neck MRI in our hospital due to tumor/nodal staging (*n* = 137), lymphadenopathy (*n* = 122), inflammatory neck lesion (*n* = 85), congenital neck lesion (*n* = 12), and patient request (*n* = 31). Among them, 375 patients were excluded because of a lack of appropriate histopathological data on the thyroid parenchyma.

**Results:** Among the patients included, 10 had normal thyroid parenchyma, 1 had Hashimoto thyroiditis, and 1 had diffuse hyperplasia. The common MRI features of normal thyroid parenchyma include iso-/slightly high and homogeneous signal intensity on T1/T2-weighted images, normal anteroposterior diameter of the thyroid gland, smooth margin, and homogeneously increased enhancement as compared to adjacent muscle. Hashimoto thyroiditis exhibited high and inhomogeneous signal intensity on T2-weighted images, while diffuse hyperplasia revealed an increased anteroposterior diameter and lobulated margin of the thyroid gland, and inhomogeneous enhancement.

**Conclusions:** MRI may be helpful for detection of incidental DTD.

## Introduction

Thyroid disease includes diffuse and nodular types (1). Diffuse thyroid disease (DTD) may be associated with thyroid dysfunction, and the thyroid function test and serology are widely used in evaluating thyroid dysfunction (2). In clinical practice, however, accurate detection of subclinical or asymptomatic DTD is not easy but this detection can be helpful for the appropriate management of thyroid dysfunction (2). Thyroid ultrasonography (US) is widely used to detect and characterize thyroid nodules (3). Although clinical and laboratory findings have played a key role in the diagnosis and treatment of DTD, the use of thyroid US for detecting DTD is feasible (4–7). Moreover, recent studies have demonstrated that computed tomography (CT) may be helpful to detect DTD (8–10). CT detection of DTD may be practical, as CT is widely used in the evaluation of neck lesions. In the literature, magnetic resonance imaging (MRI) is reported to be helpful for differentiating Graves' disease from Hashimoto thyroiditis or painless thyroiditis (11–14). Hashimoto thyroiditis and Graves' disease exhibit increased signal intensity (SI) on T1/T2-weighted images, whereas as the normal thyroid reveals somewhat greater SI than that of adjacent muscle on both images (11, 12). In addition, diffusion-weighted MRI may be helpful for differentiation between Hashimoto thyroiditis, Graves' disease, and painless thyroiditis (13, 14). However, no previous studies have investigated the feasibility of using MRI for detection of incidental DTD.

For evaluating head and neck cancer, contrast-enhanced CT is the most commonly used imaging modality (15). MRI has excellent soft tissue differentiation, but CT provides exceptional anatomical detail with greater spatial resolution than MRI (15). In particular, MRI is commonly used to determine tumor/nodal staging of head and neck cancer, to identify a primary site in patients with unknown primary head and neck cancer, and to evaluate lymphadenopathy in the neck (15). If incidental MRI detection of DTD is possible, as with US and CT, MRI may facilitate appropriate management of DTD.

Therefore, the purpose of this study was to assess the MRI features of normal thyroid parenchyma and incidental DTD.

## Materials and Methods

### Patients

This retrospective study was approved by Busan Paik Hospital institutional review board (IRB 17-0212), and requirement for obtaining informed patient consent was waived because of the retrospective nature of the investigation and the use of anonymized patient data. From January 2008 to December 2017, 387 patients (113 women, 274 men; mean age 57.1 ± 19.3 years [range 1–92 years]) underwent neck MRI in our hospital for tumor/nodal staging (*n* = 137), lymphadenopathy (*n* = 122), inflammatory neck lesion (*n* = 85), congenital neck lesion (*n* = 12), and by patient request (*n* = 31). Among them, only the cases with an available histopathological specimen of the thyroid gland, obtained by thyroid surgery, were included. In addition, the interval between neck MRI and thyroid surgery had to be less than 12 months. Ultimately, a total of 12 patients (7 women, 5 men; mean age 49.6 ± 16.2 years [range 22–70 years]) were included in the study.

### Magnetic Resonance Imaging Protocol

MRI was performed using 1.5 Tesla units (Gyroscan NT, Philips, Best, Netherlands; and Genesis Signa, GE Healthcare, Chicago, IL, United States) and 3 Tesla units (Skyra, 20-channel phased array coil, Siemens, Erlangen, Germany; and Achieva, Philips, 16-channel phased array coil). MRI at our institution involves image acquisition from the skull base to the tracheal carina. All patients were imaged using a standardized MRI protocol comprising a 3-plane localizer and turbo spin-echo imaging, involving the following sequence: axial and coronal T1-weighted images (TR/TE, 730/10, FOV 220 × 220 cm, matrix 448 × 269, slice thickness 4.0 mm, intersection gap 0.4 mm), axial T2-weighted images (TR/TE, 4530/68, FOV 220 × 220 cm, matrix 448 × 269, slice thickness 4.0 mm, intersection gap 0.4 mm), axial and coronal fat-saturated T2-weighted images, followed by contrast-enhanced axial and coronal T1-weighted images. The same sequence was performed after contrast agent administration (0.1 mL/kg of gadobenate dimeglumine, Multihance BRACCO, Milan, Italy, at 2 ml per second, followed by a 20-ml saline flush at the same rate). The first post-contrast sequence began 90 sec after the contrast medium injection, to allow sufficient contrast enhancement.

### Magnetic Resonance Imaging Analysis

A single radiologist (with 16 years of experience with thyroid US examination and neck MRI analysis, after obtaining board certification) retrospectively investigated MRI findings of the thyroid gland in the study patients, while being blinded to the US diagnosis of the thyroid gland, clinico-serological information, and the patient's medication history for DTD. The following features were investigated based on MR images, using a picture achieving and computer data system: parenchymal SI (as compared to adjacent muscle and fat; iso- [same SI at that of adjacent muscle], low [low SI relative to adjacent muscle], slightly high [higher SI than adjacent muscle but lower SI than adjacent fat], or high [same SI at that of adjacent fat]; homogeneity on T1- or T2-weighted images (homogeneous and inhomogeneous); anteroposterior diameter of the thyroid gland (normal [1–2 cm], increased [>2 cm], or decreased [ < 1 cm]); glandular margin (smooth or lobulated); degree of enhancement (as compared to adjacent muscle; iso-, decreased, or increased); and pattern of enhancement (homogeneous or inhomogeneous).

### Final Diagnosis of Diffuse Thyroid Disease and Normal Thyroid Parenchyma

Histopathological findings from the thyroid gland were retrospectively analyzed by a single pathologist. Hashimoto thyroiditis was characterized as progressive loss of thyroid follicular cells with replacement by lymphocytes and formation of germinal centers associated with fibrosis. Diffuse hyperplasia was characterized by diffuse hypertrophy and hyperplasia of follicular cells with retention of the lobular architecture and no definite nodule formation. The thyroid gland was considered to have normal thyroid parenchyma when there was no visual evidence of coexisting DTD.

## Results

For the 12 patients, the reason for thyroid surgery was suspicious thyroid malignancy in cytology. The mean interval between neck MRI and thyroid surgery was 7.6 ± 2.9 months (range 2–12 months). After thyroid surgery (9 total thyroidectomy and 3 hemithyroidectomy), papillary thyroid carcinoma was confirmed in all patients. Based on the histopathological findings of the thyroid gland, we characterized patients with normal thyroid parenchyma (*n* = 10), Hashimoto thyroiditis (*n* = 1), and diffuse hyperplasia (*n* = 1).

**Figure 1:**
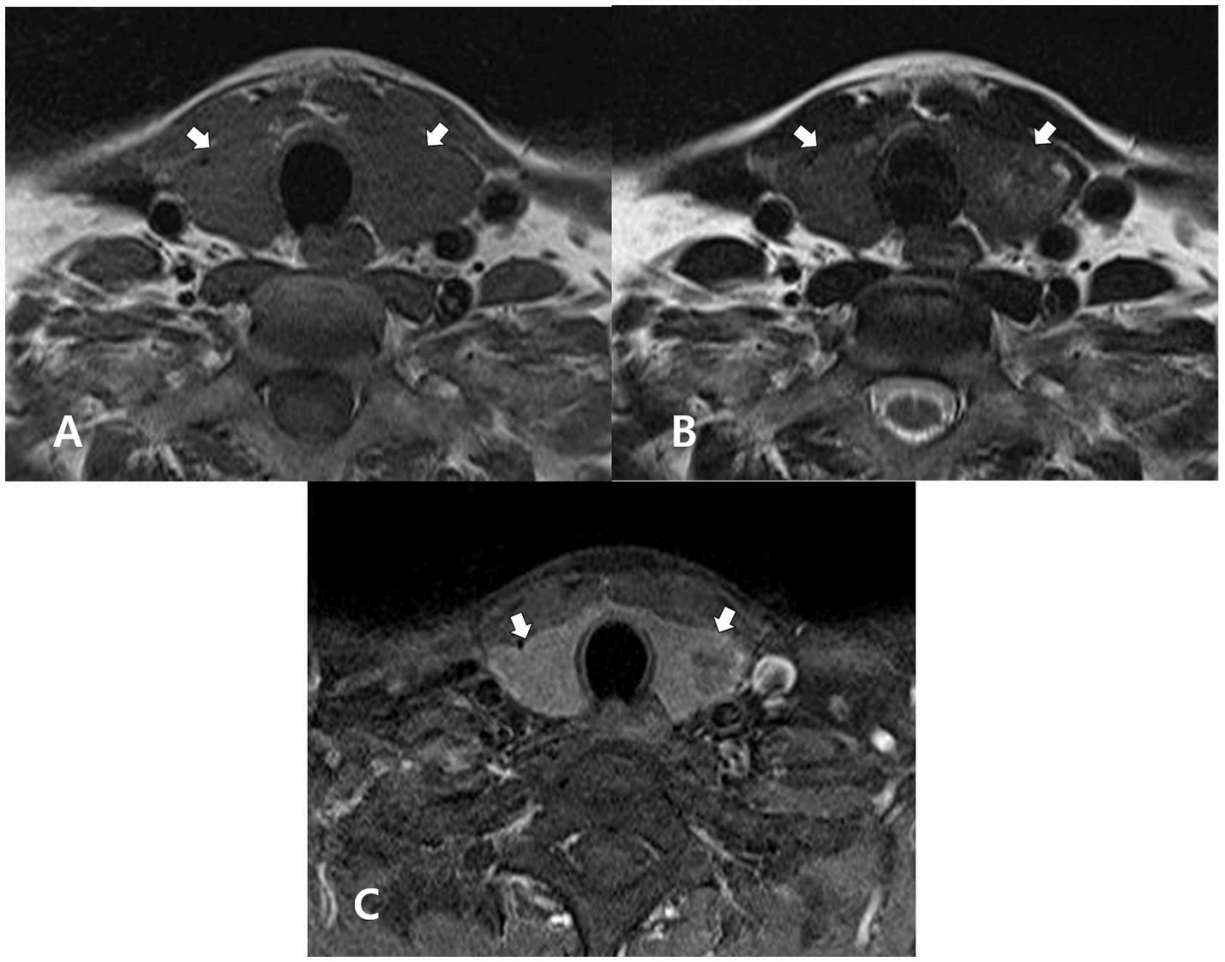
A 51-year-old man with normal thyroid parenchyma confirmed by histopathology after thyroid surgery for the treatment of papillary thyroid carcinoma. In the non-enhanced axial T1- **(A)** and T2- **(B)** weighted images, the thyroid gland (arrows) exhibits homogeneous, iso-, and slightly high signal intensities, respectively, when compared with adjacent muscle. In both images, the thyroid gland (arrows) exhibits a normal size and smooth margin. In the enhanced axial, fat-suppression T1-weighted image **(C)**, the thyroid gland (arrows) exhibits homogeneously increased enhancement, when compared with adjacent muscle.

**Figure 2:**
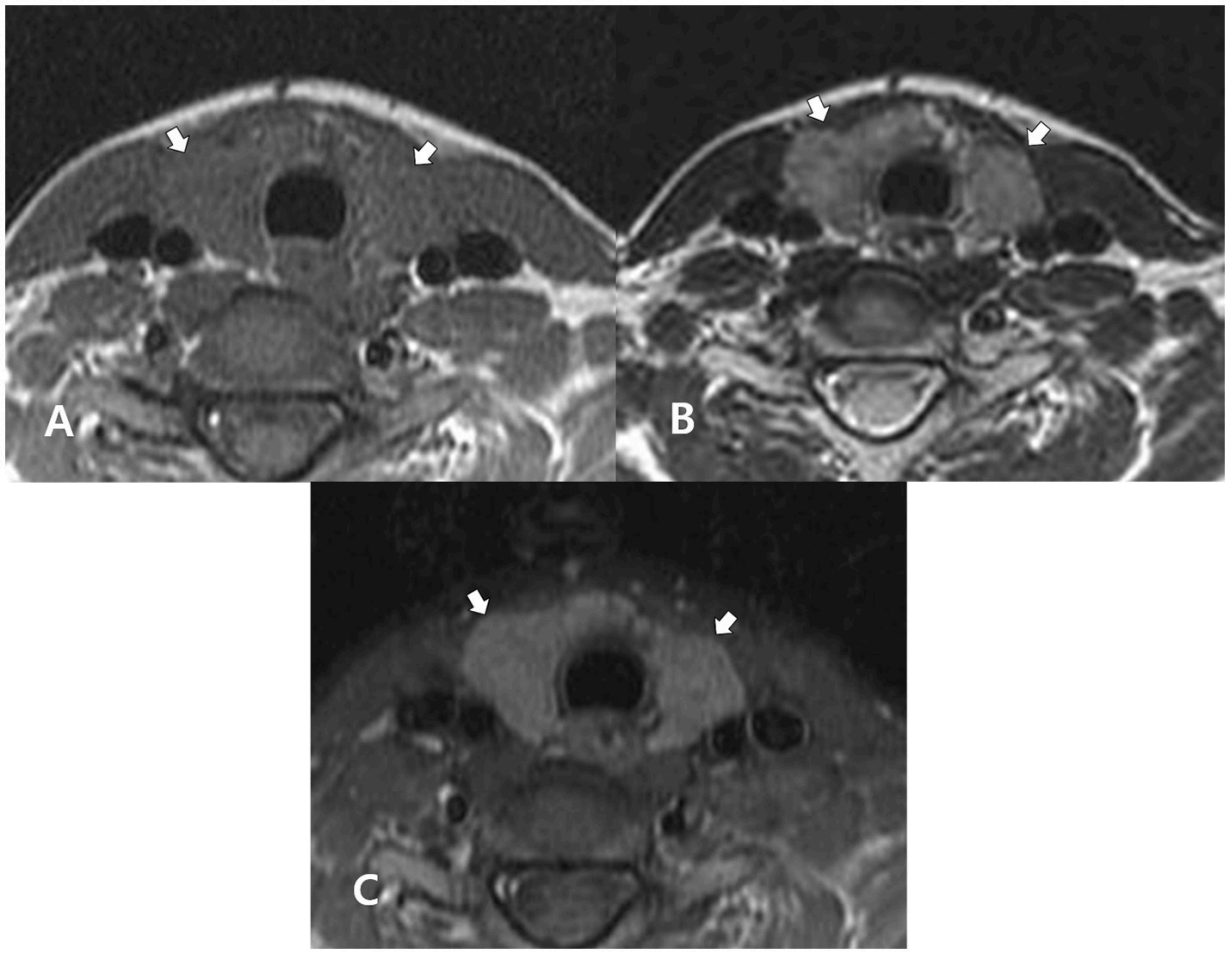
A 27-year-old woman with Hashimoto thyroiditis confirmed in cytology after ultrasound-guided fine-needle aspiration. In the non-enhanced axial T1- **(A)** and T2- **(B)** weighted images, the thyroid gland (arrows) exhibits homogeneous, iso-signal intensity, and inhomogeneous, high signal intensity, respectively, when compared with adjacent muscle. In both images, the thyroid gland (arrows) exhibits a normal gland size and smooth margin. In the enhanced axial, fat-suppression T1-weighted image **(C)**, the thyroid gland (arrows) exhibits homogeneously increased enhancement, when compared with adjacent muscle.

**Figure 3:**
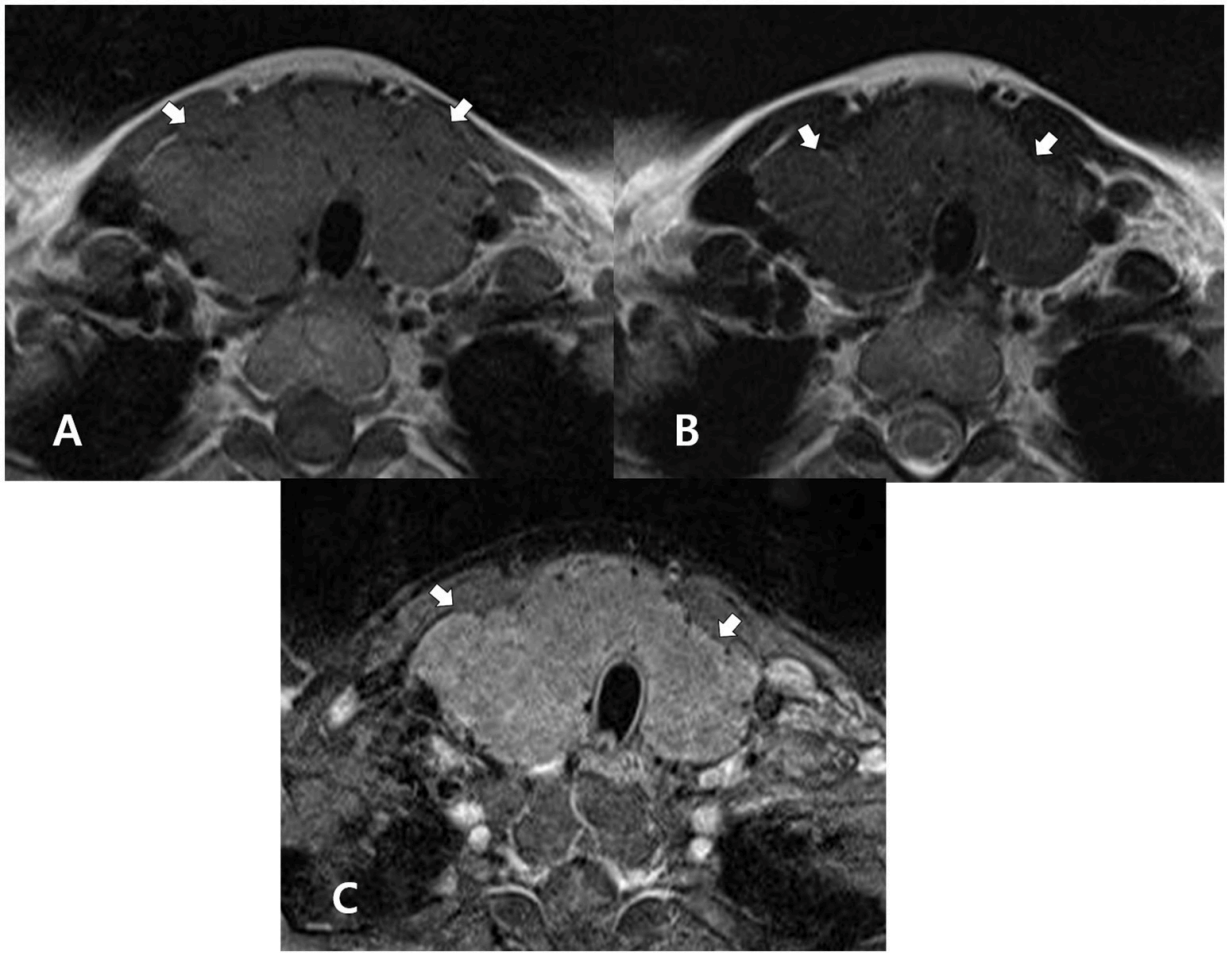
A 44-year-old woman with diffuse hyperplasia confirmed by histopathology after thyroid surgery for the treatment of papillary thyroid carcinoma. In the non-enhanced axial T1- **(A)** and T2-**(B)** weighted images, the thyroid gland (arrows) exhibits homogeneous, iso-. and slightly increased signal intensity, respectively, when compared with the adjacent muscle. In both images, the thyroid gland (arrows) exhibits increased size and a lobulated margin. In the enhanced axial, fat-suppression T1-weighted image **(C)**, the thyroid gland (arrows) exhibits increased, but inhomogeneous enhancement, when compared with adjacent muscle.

The MRI features of the thyroid gland are listed in Table 1. The 9 normal thyroid parenchyma cases exhibited iso-/slightly high SI on T1/T2-weighted images, whereas 1 case revealed iso-/slightly high SI on T1/T2-weighted images. In all normal thyroid parenchyma cases, SI was homogeneous, the anteroposterior diameter of the thyroid gland was normal, had a smooth margin, and showed homogeneously increased enhancement as compared with adjacent muscle (Figure 1). In contrast, Hashimoto thyroiditis exhibited high and inhomogeneous SI on T2-weighted images (Figure 2), and diffuse hyperplasia revealed increased anteroposterior diameter and lobulated margin of the thyroid gland and inhomogeneous enhancement (Figure 3).

**Table 1:** Comparison of magnetic resonance imaging features of normal thyroid parenchyma and diffuse thyroid disease in 12 patients.

**MRI features**	**Normal thyroid parenchyma (*n* = 10)**	**Diffuse thyroid disease (*n* = 2)**
**SI on T1 WI**		
iso-	10 (100)	2 (100)
Low	0 (0)	0 (0)
slightly high	0 (0)	0 (0)
High	0 (0)	0 (0)
**SI on T2 WI**		
iso-	1 (10)	0 (0)
Low	0 (0)	0 (0)
slightly high	9 (90)	1 (50)
High	0 (0)	1 (50)
**HOMOGENEITY on T1 or T2 WI**		
Homogeneous	10 (100)	1 (50)
Inhomogeneous	0 (0)	1 (50)
**AP DIAMETER OF THE THYROID**		
Normal	10 (100)	1 (50)
Increased	0 (0)	1 (50)
Decreased	0 (0)	0 (0)
**MARGIN OF THYROID GLAND**		
Smooth	10 (100)	1 (50)
Lobulated	0 (0)	1 (50)
**DEGREE OF ENHANCEMENT**		
iso-	0 (0)	0 (0)
Decreased	0 (0)	0 (0)
Increased	10 (100)	2 (100)
**PATTERN OF ENHANCEMENT**		
Homogeneous	10 (100)	1 (50)
Inhomogeneous	0 (0)	1 (50)

*Data presented in parentheses are percentage of each item. MRI, magnetic resonance imaging; SI, signal intensity; WI, weighted image; AP, anteroposterior*.

## Discussion

In the literature, the known US features of normal thyroid parenchyma included a fine echotexture, iso-echogenicity, a smooth margin, a normal glandular size, and normal parenchymal vascularity (4–7). In contrast, the known CT features of normal thyroid parenchyma include iso-attenuation, homogeneous attenuation, an anteroposterior diameter of 1–2 cm, smooth margin, and homogeneous enhancement (8–10). In the present MRI study, the thyroid gland was compared with adjacent muscle. The common MRI features of normal thyroid parenchyma include iso-/slightly high SI on T1/T2-weighted images, homogeneous SI, normal anteroposterior diameter of the thyroid gland, smooth margin, and homogeneously increased enhancement. According to previous MRI studies, normal thyroid parenchyma exhibits greater SI than adjacent muscle on T1-weighted images (11, 12). However, all normal thyroid parenchyma cases in the present study exhibited iso-SI on T1-weighted image. The reason for this difference is unclear. To clarify this issue, further studies are required.

In the literature, the reported US features of DTD include increased or decreased parenchymal echogenicity, coarse echotexture or “micronodulation,” increased or decreased anteroposterior diameter of the thyroid gland, presence of marginal nodularity, and increased or decreased parenchymal vascularity (4–7). In contrast, the CT features suggestive of DTD include low attenuation, inhomogeneous attenuation, increased glandular size, lobulated margin, and inhomogeneous enhancement (8–10). In the present MRI study, SI, glandular size or margin, and parenchymal enhancement may be different between normal thyroid parenchyma and DTD. Nevertheless, among the numerous studies, no US or CT features with a high sensitivity and specificity for detecting DTD have been found (4–10). Similarly, no specific MRI features for DTD were found. Nevertheless, MRI detection of DTD may be feasible, although only 2 cases of DTD were included. To clarify this issue, a large-scale study is required.

This study had several limitations. First, the sample size was small. In particular, only 2 cases of DTD were included. Secondly, a neck MRI protocol was used in this study. For clarity, a further study using the appropriate MRI protocol for the thyroid gland is required. Thirdly, there was a considerable interval between neck MRI and thyroid surgery. Fourthly, a single radiologist performed the image analysis. Finally, only patients who underwent neck MRI and thyroid surgery were included, and thus selection bias was unavoidable.

In conclusion, the results of the present study demonstrated that the MRI features of normal thyroid parenchyma and incidental DTD are different. Thus, MRI may be helpful for detection of incidental DTD.

## Ethics Statement

This study follows the principles expressed in the Declaration of Helsinki. All study participants waived informed consents owing to the retrospective analysis, and the study design was approved by the appropriate ethics review boards.

## Author Contributions

DWK concept and design. HB, DWK analysis and interpretation of data; TK, DWK manuscript writing; DWK review of final manuscript. All authors acquisition of data, literature review, and refinement of manuscript.

### Conflict of Interest Statement

The authors declare that the research was conducted in the absence of any commercial or financial relationships that could be construed as a potential conflict of interest.
